# True Prevalence of Shigellosis in Indian Children with Acute Gastroenteritis: Have We Been Missing the Diagnosis?

**Published:** 2016-02-02

**Authors:** Prabhav Aggarwal, Beena Uppal, Roumi Ghosh, Subramaniam Krishna Prakash, Anita Chakravarti, Krishnan Rajeshwari

**Affiliations:** ^a^ Department of Microbiology, Maulana Azad Medical College, Bahadur Shah Zafar Road, New Delhi-110002, India; ^b^ Department of Pediatrics, Maulana Azad Medical College and associated Lok Nayak Hospital, Bahadur Shah Zafar Road, New Delhi-110002, India

**Keywords:** *Shigella*, Diarrhea, Dysentery, *ipaH*, India

## Abstract

**Background:** *Shigella* is responsible for high morbidity and mortality among children, yet its true prevalence remains inconclusive. The aim of this study was to determine the actual prevalence of *Shigella* infection in childhood diarrhea and dysentery cases and assess the applicability of *ipaH* gene PCR in Indian settings.

**Methods:** This study was conducted at Maulana Azad Medical College and associated Lok Nayak Hospital, New Delhi, India during 2011-12. A total of 385 children (207 with diarrhea, 118 with dysentery, and 60 matched controls) were enrolled. Stool samples were cultured, and the suspected colonies were analyzed using biochemical reactions and serotyping. Antimicrobial susceptibility testing was done using disc diffusion method. *ipaH*-gene PCR was performed directly on stool samples collected from 180 randomly selected patients (60 from each group).

**Results:** *Shigella* was isolated using conventional culture methods in 8.2% (95% CI: 5.1%, 12.8%), 33.1% (95% CI: 25.2%, 42.0%), and 0% in the diarrhea, dysentery and control cases, respectively. High resistance was seen towards co-trimoxazole, nalidixic acid, fluoroquinolones, doxycycline and several beta-lactams drugs. Actual prevalence of shigellosis was determined using *ipaH* gene PCR to be 18.3% (95% CI: 10.4% – 30.1%) diarrhea cases and 56.7% (95% CI: 44.1, 68.4%) dysentery cases. One (1.7%, 95% CI: 0.01%, 9.7%) control specimen also yielded positive result in PCR.

**Conclusions:** Correct diagnosis of shigellosis is essential to start antimicrobial therapy in selected cases. The prevalence of *Shigella* / EIEC infection in children is much higher than previously estimated. Despite its high costs and other limitations, we recommend the use of *ipaH*-gene PCR as a routine tool in the management of childhood acute gastroenteritis cases.

## Introduction

*Shigella* is responsible for high morbidity and mortality in regions with poor sanitation and personal hygiene, particularly in children. An annual worldwide incidence of 164.7 million cases of shigellosis has been estimated; of them, 163.2 million cases were in the developing countries, resulting in 1.1 million deaths^1^. Sixty-nine percent of all episodes and 61% of all deaths involved children of age less than 5 yr^1^. The importance of the disease is further highlighted by the fact that diarrhea is the second most common cause of worldwide death among children under five yr of age, closely following pneumonia^2^. Unfortunately, the highest number of diarrhea-related deaths in the world is reported in India^2^.

Typically, *Shigella* infection is associated with bloody diarrhea, although some cases present only with loose stool without the presence of blood. *S. sonnei* and *S. boydii* are usually associated with mild illness of short duration, whereas *S. dysenteriae* and *S. flexneri* infections are more severe. The latter may be associated with complications, such as dehydration, growth retardation, anemia, hemolytic uremic syndrome (HUS), Reiter’s syndrome, and renal failure. Shigellosis is not yet a vaccine preventable disease^3^. Infection with enteroinvasive *Escherichia coli* (EIEC) presents with clinical features that are similar to those of shigellosis. However, EIEC infection is very infrequent as compared to shigellosis^4,5^.

Apart from *Shigella* and EIEC, diarrhea and dysentery may be caused by a large number of other infectious agents, making the specific clinical diagnosis of shigellosis difficult and emphasizing the important role of relevant and timely laboratory investigations. Traditional routine microscopic examination and stool culture are employed most frequently but are relatively inefficient, time-consuming, and labor-intensive methods. Large number of clinically suspicious cases remains undiagnosed. Specific diagnosis of shigellosis is essential as it often requires early antimicrobial therapy, in contrast to other diarrheal illnesses for which only rehydration therapy is routinely recommended which may reduce complication rates^6^.

This study was designed to determine the real prevalence of *Shigella*/EIEC in Indian childhood diarrhea and dysentery cases using PCR to detect the invasion plasmid antigen H (*ipaH*) gene sequence for direct diagnosis by analysis of stool specimens. In addition, we attempted to analyze the cost-effectiveness and applicability of this PCR technique for routine diagnosis in Indian laboratory setting.

## Methods

### Setting and sample collection

A two-year prospective study (2011-12) was designed at Maulana Azad Medical College and associated 1600-bedded Lok Nayak Hospital, New Delhi, India, involving 385 children of age less than 12 yr. This number included 207 children with acute diarrhea, 118 with acute dysentery, and 60 with no gastrointestinal complaints (age and sex matched controls).

At enrolment, informed consent was obtained from parents/guardians, who were asked to complete a questionnaire consisting of socio-demographic and personal details, history of diarrheal episodes, clinical signs and symptoms, etc. The study was approved by the institutional Ethics Committee.

Acute diarrhea was defined as increased frequency of stools with soft/liquid consistency, equal to or more than three times per day and of duration less than 14 days^7^. Children with dysentery had passage of blood in the stools, as observed by their guardian or the treating physician. Children already subjected to antimicrobial therapy and/or had other known causes of blood in the stools, such as rectal polyps, inflammatory bowel disease, bleeding diathesis, etc., were excluded from the study.

### Stool culture and antimicrobial susceptibility testing

Fecal specimens were collected from all the participants in a clean, wide mouth, screw-capped, and disposable plastic container and were transported to the Enterobacteriaceae laboratory, Department of Microbiology, Maulana Azad Medical College within two hours. In case of delay, the samples were transported in Cary Blair medium / buffered glycerol saline. They were preserved at -70 °C until molecular investigations were completed. The samples were subjected to routine microscopic examination for the presence of red blood cells, fecal leukocytes, parasitic cysts, trophozoites, helminthic ova, and larvae.

Stool samples were cultured directly onto MacConkey’s agar, Xylose Lysine Desoxycholate agar (XLD agar), and Bile salt agar (BSA) (HiMedia, Mumbai, India) and incubated overnight at 37 °C in ambient air. Specimens were also enriched for 6–8 h in selenite F broth and alkaline peptone water and plated on XLD agar and BSA, respectively. Non-lactose fermenting colonies from MacConkey’s agar and XLD agar were selected and identified based on biochemical reactions and serotyping (Denka Seiken Co., Ltd., Tokyo, Japan). EIEC were differentiated from *Shigella* based on biochemical characteristics, Vitek® 2 and serotyping. *Shigella* isolates were then subjected to antimicrobial susceptibility testing by the disc diffusion method^8^.

### ipaH-gene PCR

**Figure 1:**
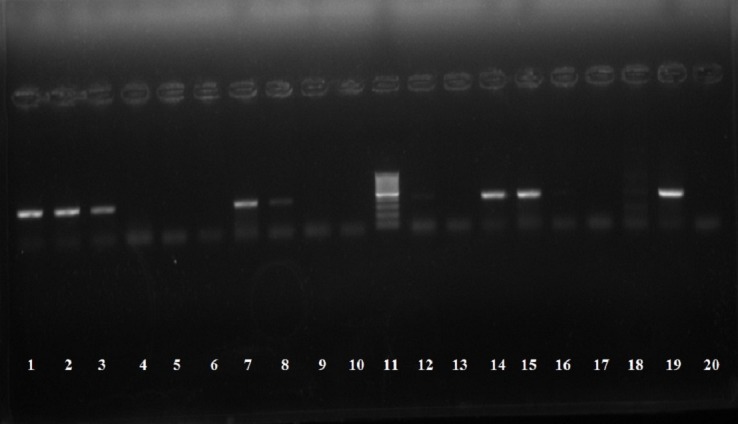
Gel electrophoresis showing amplification products of *ipaH* gene at 424 bp (Lanes 1-10 and 12-18: Test samples; Lane 11: 100bp DNA ladder; Lane 19: Positive control; Lane 20: Negative control)

Stool samples from 180 children (60 randomly selected samples from each group: diarrhea, dysentery, and control) were subjected to direct PCR to detect the presence of *ipaH* gene. DNA was extracted directly from the stool sample using the QIAamp DNA Stool Mini Kit (QIAGEN, Germany) as per the manufacturer’s instructions and used as template DNA. Amplification of a 424 bp region of *ipaH* gene was conducted using the primer sequences described previously: F[5’ GCTGGAAAAACTCAGTGCCT3’] and R[5’ CCAGTCCGTAAATTCATTCT3’]^9^. The reaction mixture (25 μL) consisted of template DNA (6 μL), 10x PCR reaction buffer, 15 mM MgCl_2_, 50 pM of each primer, 0.25 mM concentration of each of dNTP (dATP, dCTP, dNTP, and dTTP) and 1 U of Taq DNA polymerase. Thirty-five cycles of amplification were performed in a thermal cycler (Bio-Rad Laboratories Inc.), consisting of initial denaturation at 96 °C for 10 min, denaturation at 94 °C for 60 s, annealing at 56 °C for 120 s, extension at 72 °C for 60 s and final extension at 72 °C for 10 min. Positive (*S. Flexneri* ATCC® 12022) and negative controls (*E. coli* ATCC® 25922) were included in each run of PCR. Amplified PCR products were electrophoresed on 2% ethidium bromide stained agarose gel, along with 100 bp DNA ladder. The bands in the gel were visualized and photographed under UV trans-illumination (Figure 1).

Several known Gram-negative bacterial strains were also tested by the same *ipaH* PCR protocol to establish the specificity of the reaction: *Salmonella* Typhi (five strains), *S*. *paratyphi* A (three strains), *S. typhimurium* (two strains), *Pseudomonas aeruginosa* (five strains), *Klebsiella pneumoniae* (four strains), *Citrobacter freundii* (two strains), *Enterobacter cloacae* (two strains) and* E. coli* (ATCC® 25922).

### Statistical analysis

A case was considered to have *Shigella*/ EIEC infection if it was diagnosed by either one or both of the techniques used, i.e., by culture and/or PCR. Fisher’s exact test was utilized to compare the results in the diarrhea and dysentery groups; *P* value of <0.05 was considered significant. Wherever applicable, 95% confidence interval was calculated. Costs of reagents, equipment, and emoluments of trained personnel were calculated approximately as per the year 2013.

## Results

**Figure 2:**
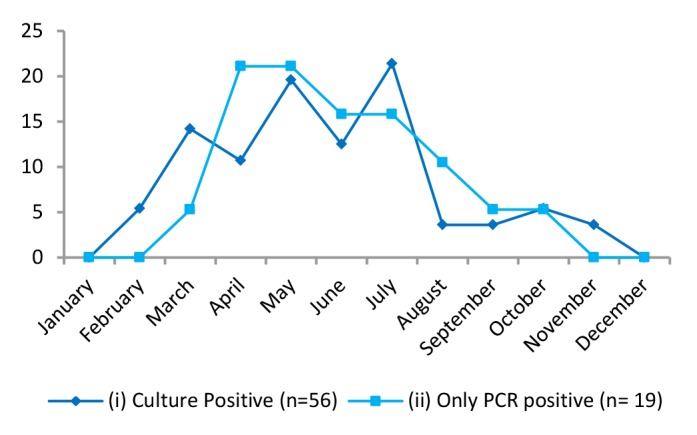
Month-wise distribution (%) of shigellosis cases, which were (i) culture positive and (ii) PCR positive, culture negative* Shigella* spp.

During the two-year long study period, 207 children with acute diarrhea, 118 children with acute dysentery, and 60 controls met inclusion and exclusion criteria. The median age for the diarrhea, dysentery, and control groups were 18, 15, and 18 months, respectively. All groups had a slight male predominance, with a male: female ratio ranging from 1.07 to 1.22. Maximum number of diarrhea and dysentery cases was reported during summers and monsoons. Seasonal distribution of culture identified cases as well as *ipaH* specific PCR-positive (but culture-negative) cases were similar, with maximum number of cases observed during the months of March to July (Figure 2).

At the time of presentation, fever, vomiting, and abdominal pain were the most common symptoms in the dysentery group. Failure to pass urine in the preceding eight hours was established more frequently in children with diarrhea (*P*<0.001). While all patients with dysentery had history of passage of blood in the stools, only 90.7% (107/118) of the dysentery stool samples submitted to the laboratory contained visible blood. As per the definition, none of the samples from diarrhea patients contained visible blood (Table 1). A total of 12 specimens showed presence of parasitic ova and/or cysts (*Entamoeba histolytica*, *Giardia lamblia*, *Ascaris lumbricoides*, and *Hymenolepis nana)* on microscopy.

**Table 1:** Clinical and investigational findings of diarrhea, dysentery, and control cases

**Variables**	**Diarrhea** **n= 207 (%)**	**Dysentery** **n=118 (%)**	**Control** **n=60 (%)**	***P*** ** value**
Gender				0.729
Male	107 (51.7)	64 (54.2)	33 (55.0)	
Female	100 (48.3)	54 (45.8)	27 (45.0)	
Having fever				0.001
Yes	51 (26.6)	70 (59.3)	0 (0.0)	
No	156 (73.4)	48 (40.7)	60 (100)	
Having vomiting				0.129
Yes	31 (14.9)	26 (22.0)	0 (0)	
No	176 (85.1)	92 (78.0)	60 (100)	
Having abdominal pain				0.001
Yes	15 (7.2)	37 (31.4)	0 (0.0)	
No	192 (92.8)	81 (68.6)	60 (100)	
Pass urine in preceding 8 hours				0.001
Yes	60 (29.8)	2 (1.7)	1 (1.7)	
No	147 (70.2)	116 (98.3)	59 (98.3)	
Having irritability				0.022
Yes	27 (13.0)	6 (5.1)	0 (0.0)	
No	180 (87)	112 (94.9)	60 (100)	
Having lethargy				0.022
Yes	16 (7.7)	2 (1.7)	0 (0.0)	
No	191 (92.3)	116 (98.3)	60 (100)	
Passage of blood in stools				0.001
Yes	0 (0.0)	118 (100)	0 (0.0)	
No	207 (100)	0 (0.0)	60 (100)	
Visible blood on gross examination				0.001
Yes	0 (0.0)	107 (90.7)	0 (0.0)	
No	207 (100)	11 (9.3)	60 (100)	
White blood cell/ HPF				0.001
≥10	37 (17.8)	81 (68.6)	1 (1.7)	
<10	170 (82.2)	37 (31.4)	59 (98.3)	
Culture for *Shigella*				0.001
Positive	17 (8.2)	39 (33.1)	0 (0.0)	
Negative	190 (91.8)	79 (66.9)	60 (100)	
PCR				0.001
Positive	11/60 (18.3)	34/60 (56.7)	1/60 (1.7)	
Negative	49/60 (81.7)	26/60 (43.3)	59/60 (97.3)	

*Shigella* spp. could be isolated by standard microbiological culture in 17 of the 207 diarrheal cases (8.2%, 95% CI: 5.1%, 12.8%) and 39 of the 118 dysentery cases (33.1%, 95% CI: 25.2%, 42.0%) (Table 1). None of the control group samples yielded *Shigella* on culture. Species distribution was similar in both groups; *S. flexneri* (66.1%) was the most common, followed by *S. boydii* (24.1%) and *S. sonnei* (12.5%). *S. dysenteriae* was not isolated during the study period. One strain of EIEC was also isolated from the dysentery group.

*Shigella* showed high resistance to several antimicrobial agents, including co-trimoxazole (92.8%), nalidixic acid (91.1%), doxycycline (87.5%), ampicillin (58.9%), ofloxacin (57.1%), and ciprofloxacin (53.6%). Among the aminoglycosides tested, resistance was higher against gentamicin (10.7%) as compared to amikacin (3.6%). Sixteen percent of the strains were resistant to the third generation cephalosporins (ceftazidime, ceftriaxone, and cefotaxime). All the strains, however, remained susceptible to the carbapenems.

Notably, PCR was positive in all of the stool samples yielding *Shigella* or EIEC on culture (Table 2). Overall, prevalence of shigellosis was determined by *ipaH* gene PCR to be 18.3% (95% CI: 10.4%, 30.1%) diarrhea cases and 56.7% (95% CI: 44.1, 68.4%) dysentery cases (Figure 1). One of the 60 (1.7%, 95% CI: 0.01%, 9.7%) control specimens also yielded positive results in PCR.

**Table 2:** Relationship between *Shigella*/EIEC identified by culture and *ipaH* PCR in stool samples from diarrhea, dysentery, and control cases

**Variables**	**PCR positive**	**PCR negative**	**Total**
Diarrhea (n=60)			
Culture positive	5	0	5
Culture negative	6	49	55
Total	11	49	60
Dysentery (n=60)			
Culture positive	22	0	22
Culture negative	12	26	38
Total	34	26	60
Control subjects (n=60)			
Culture positive	0	0	0
Culture negative	1	59	60
Total	1	59	60

Sensitivity of culture method, while considering PCR as the gold standard was 45.4% and 68.1% for the diarrhea and dysentery groups, respectively; while the specificity was 100% for both the groups.

## Discussion

Analysis of the epidemiological data showed that a large proportion of patients belonged to the group of very young age, with 86.8% of the enrolled symptomatic children being less than five years of age. Similarly, 43 of the 56 (76.8%) *Shigella* strains were isolated from children less than five years of age. These results correlate with previous findings, indicating higher morbidity among young children^1^.In our study, symptoms of patients presenting with dysentery and diarrhea were different as expected; fever, vomiting, and abdominal pain were reported more frequently in children with dysentery. Previous Indian studies from Delhi^10^,West Bengal^9,11^and Karnataka^12^have reported *S. flexneri* as most common species. This is consistent with the results of the present study.

Epidemiological studies performed to estimate the prevalence of shigellosis are usually based on routine culture techniques. A literature review of sources published in the period from 1966 to 1997 suggested that *Shigella* spp. may be responsible for 3.2% to 22% of the cases of diarrhea in children aged from 0 to 14 yr^1^. Previous studies from India have reported isolation of *Shigella* spp. in 3.58% to 7.7% diarrhea cases in various age groups^9,12-14^.

Our study highlights that the use of molecular methods reveals the true prevalence of *Shigella* infection, which turns out to be much higher than previously estimated. The prevalence increased from 8.2% to 18.3% in the diarrhea group and from 33.1% to 56.7% in the dysentery group, when diagnosed by PCR; indicating that approximately, half of the cases are being routinely missed.

*ipaH* gene based PCR has been reported to have high sensitivity and specificity for diagnosis of shigellosis by several studies^9,15^.Studies determining the prevalence of *Shigella* using this PCR in acute childhood diarrhea and dysentery from India and all over the world are scarce. From Vietnam, Thiem et al. reported 61% prevalence of shigellosis in children below 5 years of age who presented with dysentery^15^.Similarly Lindsay et al. reported increased prevalence using similar PCR^16^.While from West Bengal in India, Duttta et al., reported an increase in detection rates from 7.7% to 15.3% by *ipaH* gene PCR; however, they did not distinguish between children with diarrhea and dysentery^9^.

In order to rule out the possibility of false positive results due to procedural cross-contamination, healthy controls were included in the present study. PCR was positive in only one (1.7%) of the control samples, which might have been caused by brief carriage^16^.Apart from *Shigella*, *ipaH* gene is known to be present in EIEC^9^. However, EIEC is rarely isolated and not expected to cause significant alteration in the results. This is similar to our findings, wherein, we isolated only one EIEC strain, in contrast to the 56 isolates of *Shigella*^4,5^.

Culture performed poorly with respect to *ipaH* gene PCR for diagnosis of shigellosis, with very low sensitivities for both diarrhea and dysentery groups. Other researchers have also demonstrated similar finding, the sensitivity of culture, while taking PCR as the gold standard, being 72% and 72.9%^18,19^.Several factors may be responsible for poor sensitivity of culture technique in comparison to PCR, such as the presence of a low number of organisms in the stool sample, competition with gut flora, inappropriate sample collection, and delay in processing. In addition, if the sample is collected after the initiation of antibiotic therapy, the growth of the organisms may be hampered due to sub-lethal injuries.

The advantages of PCR in this setting are obvious. The improved rate of detection of *Shigella* will facilitate the initiation of early and appropriate antimicrobial therapy in children based on prevalent susceptibility patterns, which may reduce the duration and severity of illnesses as well as fecal shedding of these bacteria^6^. While culture methods take 48–72 h for isolation and identification, detection by PCR is possible within only few hours. Determination of the real prevalence of shigellosis (which turns out to be much higher than previous estimates) will in turn stimulate a greater focus on the disease and its management, providing impetus to the development of *Shigella* vaccines that has been lagging behind^3^. As seen in our study as well as other previous studies, the antimicrobial resistance among *Shigella* is on the rise, therefore, correct diagnosis and initiation of appropriate empiric therapy would help curb this ever growing problem^12,13,20^.

However, *ipaH* gene-based PCR is not without disadvantages. Firstly, species identification and distinction between *Shigella* and EIEC is not possible by this PCR technique; multiplex PCR from stool sample for limited speciation have been developed but are still in the evaluation stage and are associated with much higher costs^21^. Secondly, for performing antimicrobial susceptibility tests, culture isolation is mandatory. In addition, the possibility of the presence of other enteropathogens cannot be ruled out; thus, routine microscopic examination and culture would still be required. Usually done in batches, PCR has to wait for the collection of a sufficient number of samples. Then, high costs and expertise required for performance associated are often forbidding.

The cost of the initial equipment for conventional PCR generally reaches approximately INR 1 million (USD 17,000 approx.) in addition to the requirements for space and other logistic limitations. Depending upon the size and workload of a laboratory, more than one technical expert needs to be hired, which may cost approximately USD 1000 per person per month. Then, the running costs, including those for reagents, maintenance, etc., would require another INR 1000 per test (USD 15 approx.). These costs are often prohibiting in Indian settings. On the other hand, sample processing by traditional microscopy and culture can be completed at an approximate running cost of less than INR 150 per test (USD 2.5 approx.). As a result, very few centers are able to provide PCR for routine diagnosis, that too usually for diseases other than *Shigella*. However, these high costs can be offset when the money saved is considered in terms of savings made by preventing the person-hours lost and the costs for unnecessary antimicrobial therapy and prolonged hospital stay.

Considering the numerous pros and cons, at present, *ipaH*-gene PCR is recommended for a large high-throughput laboratory, in addition to conventional culture and sensitivity testing. A central lab may be designed that receives samples from several associated health-care centers, which may help to lower the cost per sample. Commercial PCR kits are also available now for detection of *Shigella* and enteropathogenic *E. coli* (Biotecon Diagnostics GmbH, Germany; Bioingentech, Chile; AmpliSens, Slovak Republic, etc.). At present, these reagents are too expensive for routine use in developing countries like India.

## Conclusions

The prevalence of *Shigella*/EIEC infection in Indian childhood diarrhea and dysentery is much higher than the rate suggested by previous epidemiological studies. Diagnosis of shigellosis calls for an antimicrobial therapy regimen, in contrast to most of the other causes. Blanket antibiotic treatment for all diarrhea and dysentery, often practiced, gives rise to high resistance as seen in the present study. Therefore, correct diagnosis of shigellosis and antibiotic treatment of selected cases is the way forward. Attributes, such as high sensitivity and rapid results make *ipaH-*gene PCR recommendable as a useful tool in routine management of childhood acute gastroenteritis cases.

## Acknowledgments

The authors would like to thank Mr. Anzar Ashraf for help in execution of PCR.

## Conflict of interest statement

None declared.
